# The impact of Exergames on emotional experience: a systematic review

**DOI:** 10.3389/fpubh.2023.1209520

**Published:** 2023-09-07

**Authors:** Lucas Murrins Marques, Pedro Makoto Uchida, Sara Pinto Barbosa

**Affiliations:** ^1^Faculdade de Medicina, Instituto de Medicina Física e Reabilitação, Hospital das Clínicas HCFMUSP, Universidade de São Paulo, São Paulo, Brazil; ^2^Pontifícia Universidade Católica de Campinas, Campinas, Brazil

**Keywords:** gamification, Exergame, emotion, emotion regulation, public health, health promotion

## Abstract

**Background:**

Gamification has proven to be a significant tool for health promotion, with a particular focus on physical activities such as Exergames, which improve not only physical, but also cognitive health. However, it is still not clear what effect the practice of Exergames has on changing the emotional experience.

**Purpose:**

The objective of this systematic review is to evaluate the impact of Exergames training on emotional experience.

**Methods:**

A systematic search was conducted in the PUBMED and SCOPUS databases. The relevant articles were screened independently by three researchers. Data concerning emotional measures and Exergame practice were extracted for analysis.

**Results:**

The search yielded 38 articles, of which 16 were included. Exergames were found to significantly impact happiness, anxiety, depressive symptoms, mental health-related quality of life, self-worth, self-esteem, self-efficacy, perceived behavioral control, vigor, vitality, intrinsic motivation, perceived energy, and relaxation.

**Conclusion:**

Our review supports the evidence that the practice of physical activity through Exergames, on the emotional experience generally generates an increase in positive emotions. In this sense, the results found support both the use of Exergames as a leisure activity that promotes wellbeing and emotional regulation, as well as for health promotion, public health, and clinical practice purposes. Our review strongly supports the notion that engaging in physical activity through Exergames generally leads to an increase in positive emotions. As a result, these findings endorse the utilization of Exergames as a leisure activity to promote well-being and emotional regulation. Moreover, Exergames hold potential for health promotion, public health, and clinical practice purposes.

## Introduction

Gamification is the use of game design elements in non-game contexts to motivate and engage individuals (1). In recent years, gamification has gained significant attention in the field of health promotion due to its potential to increase participation and improve health outcomes for patients with cancer (2), cardiovascular disease (3), rehabilitation (4), mental health needs (5, 6) and more recently, in response to the COVID-19 pandemic (7, 8).

One of the key benefits of gamification is its ability to increase motivation and engagement (9, 10). Gamification creates a sense of enjoyment and challenge, making health-promoting behaviors more appealing and desirable. By using gamification techniques such as points, badges, and leaderboards, individuals are encouraged to engage regularly in health-promoting behaviors in a fun way (11, 12). This leads to increased participation, and ultimately, improved health outcomes, such as improved balance and mobility (13). Another benefit of gamification in health promotion is the potential to increase knowledge and awareness. Also, games can be designed to provide educational content, making it easier for individuals to understand health concepts and apply them in their daily lives (14). By making health education interactive and engaging, individuals are more likely to retain the information and make positive changes to their behaviors.

Leaderboards and other forms of competition can be used to motivate individuals to engage in healthy behaviors and achieve their goals. Social support can be provided through online communities and forums where individuals can share their experiences and receive encouragement from others. Furthermore, gamification can be used to promote adherence to treatment plans, especially concerning medication adherence (15). By creating a game-like experience which rewards individuals for adhering to their treatment plans, gamification can increase medication adherence, appointment attendance, and overall treatment compliance (16). Thus, gamification has the potential to revolutionize the field of health promotion by increasing motivation, engagement, knowledge, treatment plan adherence, leading to overall well-being (17). As gamification gains popularity, it becomes essential for health professionals to grasp and employ this powerful tool in their endeavors to promote health and prevent disease.

### Impact of emotions on exercises and game-related exercises

The impact of emotions on exercise activities has been well-documented in the field of sports psychology (18). However, with the rise of Exergames, or video games that incorporate physical activity, the role of emotions in exercise has taken on a new dimension.

One of the most notable influences of emotions on exercise, especially in the context of Exergames, is motivation. Exergames, also known as video games based on physical activity, offer an effective means to boost motivation for exercise by making physical activity more enjoyable and entertaining (19). Positive emotions such as excitement, anticipation, and enthusiasm can increase motivation to engage in Exergames and maintain a consistent exercise routine (20). However, negative emotions such as boredom and frustration can decrease motivation to exercise and lead to a lack of interest in Exergames (21). In addition to motivation, emotions can significantly influence exercise performance in Exergames through the perception of effort (22) Indeed, positive emotions can lead to increased exercise performance and a willingness to sustain the activity. Conversely, negative emotions can heighten the perception of effort, making Exergames feel more challenging and less enjoyable (23). Emotions also influence exercise in Exergames through their effects on social support (24). Exergames can foster healthy competition and provide social support, even for post-stroke patients (25). Exergames frequently incorporate social features, such as multiplayer modes and online communities, which offer social support and motivation and which may also help to address loneliness (26). Positive emotions, such as camaraderie and friendship, can foster social support and promote increased exercise adherence (27). Conversely, negative emotions, like competition and jealousy, may hinder social support and lead to decreased exercise adherence (28). Emotions play a significant role in exercise activities, including Exergames. Understanding the influence of emotions in exercise can enable individuals and professionals to tailor exercise programs, including Exergames, to meet individual needs and optimize exercise outcomes. Leveraging the positive impact of emotions, such as motivation, engagement, and social support, enhances the effectiveness of Exergames as a tool for exercise and health promotion.

### Exergames and emotions

On the other hand, it is essential to consider that, besides emotions influencing gaming-related physical activity, gaming can also impact the emotional experience. Exergames, in particular, have the potential to enhance emotional well-being through the release of endorphins. Endorphins are neurotransmitters in the brain that induce feelings of pleasure and alleviate sensations of pain. Exercise, including activities like Exergames, can stimulate the release of endorphins, contributing to positive emotional experiences (29). When individuals engage in physical activity, the body releases endorphins, which can trigger feelings of happiness and euphoria (30). Exergames, which combine physical activity with engaging gameplay, can produce this same effect. In addition to the release of endorphins, Exergames can also help reduce stress and anxiety. Research has indicated that physical activity can lower cortisol levels, the stress hormone, in the body (31). By engaging in regular Exergame sessions, individuals may experience a reduction in stress and anxiety levels, leading to a more positive mood. Exergames can also be a helpful tool for those dealing with depression (32). Depression often leads to a lack of motivation and decreased physical activity, which can perpetuate negative feelings (33). Exergames provide a fun and engaging way to get active and boost physical activity levels. Furthermore, the social aspect of numerous Exergames, such as multiplayer modes or online communities, can foster a sense of connection and support.

Finally, Exergames can be a beneficial tool for children with emotional and behavioral disorders. Many children with these conditions face challenges in social interaction and emotion regulation. Gamification could be a valuable tool to assist them in recognizing emotions and developing essential emotional skills (34). Exergames offer a safe and enjoyable avenue for children with social and emotional challenges to participate in physical activity while simultaneously enhancing social skills and emotional regulation. The potential benefits of Exergames on emotional well-being are diverse, as they release endorphins, alleviate stress and anxiety, aid in managing depression, and foster improved social skills in children. Consequently, Exergames prove to be a vital tool in promoting overall health and well-being. Considering the significant number of studies associating gamification and emotional aspects, it is crucial to thoroughly investigate the emotional impact of engaging in Exergames. The present review enabled us to gain a better understanding and explore potential areas where Exergames can serve as a higher-quality psychological intervention, ultimately leading to improved mental health prospects for patients. The primary objective of this systematic review is to highlight the key studies that link Exergame practice with emotional experience modulation. Our focus is not to advocate for a specific game or prescribe a particular training method, but rather to comprehend the potential consequences of Exergame practice on emotional experiences. By doing so, we aim to shed light on the plausible emotional benefits that can be expected from engaging in Exergames.

## Methods

### Literature search

A systematic search was performed in the SCOPUS and PubMed databases, utilizing the following keywords: “Exergame” and “Emotion” or “Emotional,” specifically targeting the titles, abstracts, or keywords of the studies. The last search update was conducted between March 18th and March 20th, 2023, to ensure the inclusion of the most recent and up-to-date studies related to Exergames and emotional aspects. No additional filters, such as publication year, were applied during the search process. Furthermore, a manual search was carried out to explore other potential articles by reviewing the references identified in the individual articles, thus ensuring a comprehensive and exhaustive examination of relevant literature on Exergames and emotional aspects.

### Literature selection: inclusion and exclusion criteria

All original studies that reported the modulation of emotional experience due to Exergames intervention were included. Inclusion was restricted to articles written in English. Consequently, articles with the following characteristics were excluded: (i) perspective articles; (ii) case reports; (iii) study protocols; (iv) review articles; (v) conference papers; (vi) those lacking emotional measures; (vii) those focusing on exercise interventions other than Exergames; (viii) those written in languages other than English. Duplicated records were removed, and all titles and abstracts were screened by three authors, following the pre-specified framework and selection criteria. After the title and abstract selection, the full text of selected reports was sought and analyzed. Discrepancies were resolved through consensus among all authors.

### Quality assessment

To assess the quality of the included studies, a list of 10 criteria was created for assessment in each article: (i) Relevance of the topic; (ii) Methodological quality; (iii) Sample size; (iv) Statistical analysis; (v) Reliability and validity of measurements; (vi) Control of confounding variables; (vii) Reproducibility and consistency; (viii) Journal quality; (ix) Conflict of interests; (x) Practical and theoretical implications. Subsequently, a score between 0 and 10 points was assigned to each of the 10 criteria by two authors, with higher values indicating greater quality for that criterion. For articles that omitted certain topics, such as no mention of conflicts of interests, the score was set to zero. A mean value for each criterion for each article was then calculated, followed by a final value relative to the sum of the mean values of each criterion. This process was utilized to assign a final score to each article, ranging from 0 to 100 points.

**Figure 1:**
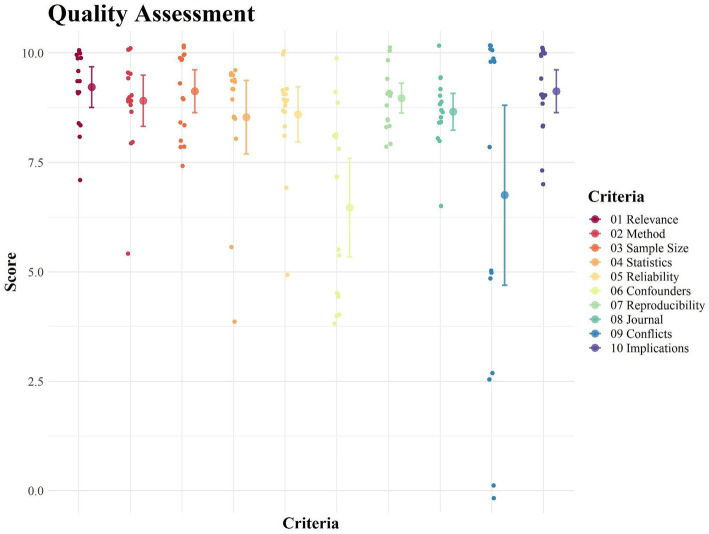
Quality assessment considering the 10 criteria.

The objective of creating this quality assessment measure was solely to establish a visual reference (as represented in Figure 1) that illustrates the possible differences between the included studies. Furthermore, considering the limited number of works, no intention was made to conduct statistical exploration or to extract any statistical inference from the differences between the articles for each criterion, nor to establish possible associations between the criteria.

### Data extraction

After a detailed review of the articles, the most significant data concerning Exergames intervention and emotional measures were identified and collected by the authors. These data were collected and independently reviewed by three authors. Subsequently, a list of variables was structured to extract key information for defining the impact of Exergames on emotional experience:

#### Sample and experimental design

(i) Sample size; (ii) Country; (iii) Characteristics defining the studied group; (iv) The study design adopted.

#### Interventions and emotional evaluation

(i) Type of intervention by exergame; (ii) Measures used to evaluate the emotional experience.

#### Observed effect

(i) Observed results of exergame intervention on emotional experience.

## Results

### Studies retrieval

**Figure 2:**
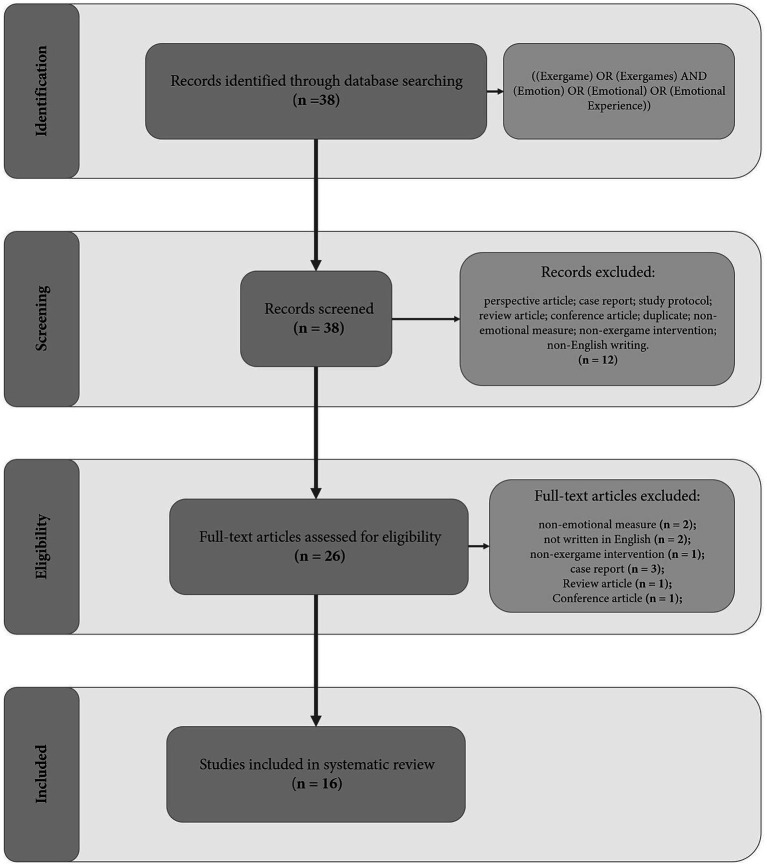
Flow diagram.

The results of the search strategy are summarized in Figure 2, presented as a PRISMA statement flow diagram (35). The literature search yielded 38 articles. Based on the screening of titles and abstracts, 12 articles were excluded. The remaining 26 articles were then assessed by reading the full text. In this phase, 10 articles were excluded as they did not meet one or more of the following criteria: (i) non-emotional measure - two articles; (ii) not written in English - two articles; (iii) exercise intervention but not exergame - one article; (iv) case report - three articles; (v) review article - one article; (vi) conference article - one article. Finally, 16 articles were included (Table 1).

**Table 1:** Summary of studies included in the review.

Study	Country	Sample size	Group	study design	Intervention length	Exercise duration	Weekly sessions	Exergame	Emotional measure	Outcome
Rosenberg et al. (36)	EUA	19	Depressive older adults	RCT	12	35 min	1	Nintendo - Wii: “Sports”	Depression, Anxiety, Health-Related QoL	Improvement in depressive symptoms, mental health-related quality of life, but not physical health-related quality of life.
Lwin & Malik (37)	Singapore	398	Healthy children	RCT	6	45 min	1	Nintendo - Wii: “Physical Exercise”	Attitude, Self-efficacy, Perceived Behavioral Control	Improvement in Attitude, Self-efficacy, and Perceived behavioral control.
Li et al. (38)	Singapore	49	Subthreshold Depressive patients	RCT	6	1 h	1	Nintendo - Wii: “Bowling and Wii Golf”	Patient Health	Improvement in subthreshold Depression; Positive emotions; and Self-efficacy.
Huang et al. (39)	Taiwan	337	Healthy adults	RCT	2	30 min	1	Microsoft - Xbox 360: ‘Your Shape: Fitness Evolved’	Vigor, Happiness	Improvement on Vigor and Happiness.
Schumacher et al. (40)	Germany	42	Hematopoietic Stem Cell Transplantation patients	Prospective randomized study	1	30 min	5	Nintendo - Wii: “Sports”	Depression, Anxiety	Improvement in Depression and Anxiety.
Nguyen et al. (2018)	Taiwan	337	Healthy children	RCT	2	30 min	1	Microsoft - Xbox 360: ‘Your Shape: Fitness Evolved’	Happiness, Optimism	Improvement in Happiness (moderated by weight control but not by optimism).
Samendinger et al. (41)	EUA	85	Healthy adults	RCT	1	30 min	6	Software-generated - “Cycling”	Motivation	Participants’ Enjoyment and Self-efficacy could predict the degree of Motivation.
Tollár et al. (42)	EUA	74	Parkinson’s patients	RCT	5	1 h	5	Microsoft - Xbox 360: ‘Just dance’; ‘Kinect Adventures video game’	Depression, Health-Related QoL	Improvement in Depression and Health-related QoL.
(43)	Taiwan	337	Healthy adults	RCT	2	30 min	1	Microsoft - Xbox 360: ‘Your Shape Fitness Evolved’	Happiness, Perceived energy, Relaxation	Improvement in Happiness, Perceived energy, and Relaxation.
Wan Yunus et al. (44)	Malaysia	36	Healthy adults	RCT	6	30 min	3	Microsoft - Xbox 360: ‘Kinect Sports’	Depression, Anxiety, Stress	Improvement in Stress.
Tollár et al. (45)	EUA	68	Multiple Sclerosis	RCT	5	1 h	5	Microsoft - Xbox 360: ‘Kinect Adventures’	Depression, Health-Related QoL	Improvement in Depression and Health-related QoL.
Zhou et al. (46)	Qatar	73	Hemodialysis patients	RCT	4	30 min	3	BioSensics - “LEGSys”	Depression	Improvement in Depression.
Andrade et al. (47)	Brazil	140	Healthy children	RCT	2	less than 40 min	1–2	Microsoft - Xbox 360: ‘Just dance’	Mood, Self-esteem	Improvement in Tension, Anger, Vigor and Self-esteem.
Deutsch et al. (48)	USA	15	Post-stroke patients	RCT	1	8.5 min	1	Microsoft - Xbox 360: ‘Kinect Light Race”	Enjoyment	Improvement in Enjoyment.
Drazich et al. (49)	USA	14	Healthy older adults	Qualitative descriptive	5	1 h	3	KATA Design Studio - “I am Dolphin”	Subjective Well Being	Improvement in Wellbeing.
Seo et al. (50)	South Korea	70	Overweight adults	RCT	8	50 min	3–5	M2Me Co. - “VRFit Cycle Pop”	Depression	Improvement in Depression.

### Demographic findings

From the information presented in Table 1, three data points related to the characterization of the studied samples were extracted, specifically: (i) country; (ii) sample size and; (iii) which group characterized the study sample.

In relation to the country of the study center, the distribution was as follows: Brazil - one article; Germany – one article; Malaysia – one article; Qatar – one article; Singapore – two articles; South Korea – one article; Taiwan – one article; USA – six articles. Concerning the sample size, the articles collectively presented a mean sample of 130.88 participants (SD = 136.25), ranging from a minimum of 14 to a maximum of 398 participants. Finally, with respect to the characteristics of the groups studied, the following were observed: Healthy adults – five articles; Healthy children – three articles; Healthy older adults – two articles; Hematopoietic Stem Cell Transplantation patients – one article; Hemodialysis patients – one article; Parkinson’s patients – one article; Older adults with Subsyndromal Depression – one article; Overweight adults – one article; Post-stroke patients – one article.

### Study design and technical aspects

Of the 16 articles, 14 (87.5%) were Randomized Clinical Trials (RCTs); the remaining included one prospective randomized study and one qualitative descriptive study.

Regarding the Exergame used, the studies intervention were the following: BioSensics - “*LEGSys*” - one article; KATA Design Studio - “*I am Dolphin*” - one article; M2Me Co. - “*VRFit Cycle Pop*” - one article; Microsoft - Xbox 360: “*Just dance*” - three articles; Microsoft - Xbox 360: “*Kinect Adventures*” - one article; Microsoft - Xbox 360: “*Kinect Light Race*” - one article; Microsoft - Xbox 360: “*Kinect Sports*” - one article; Microsoft - Xbox 360: “*Your Shape: Fitness Evolved*” - two articles; Nintendo - Wii: “*Bowling and Wii Golf*” - one article; Nintendo - Wii: “*Physical Exercise*” - one article; Nintendo - Wii: “*Sports*” - two articles; Software-generated - “*Cycling*” - one article.

**Figure 3:**
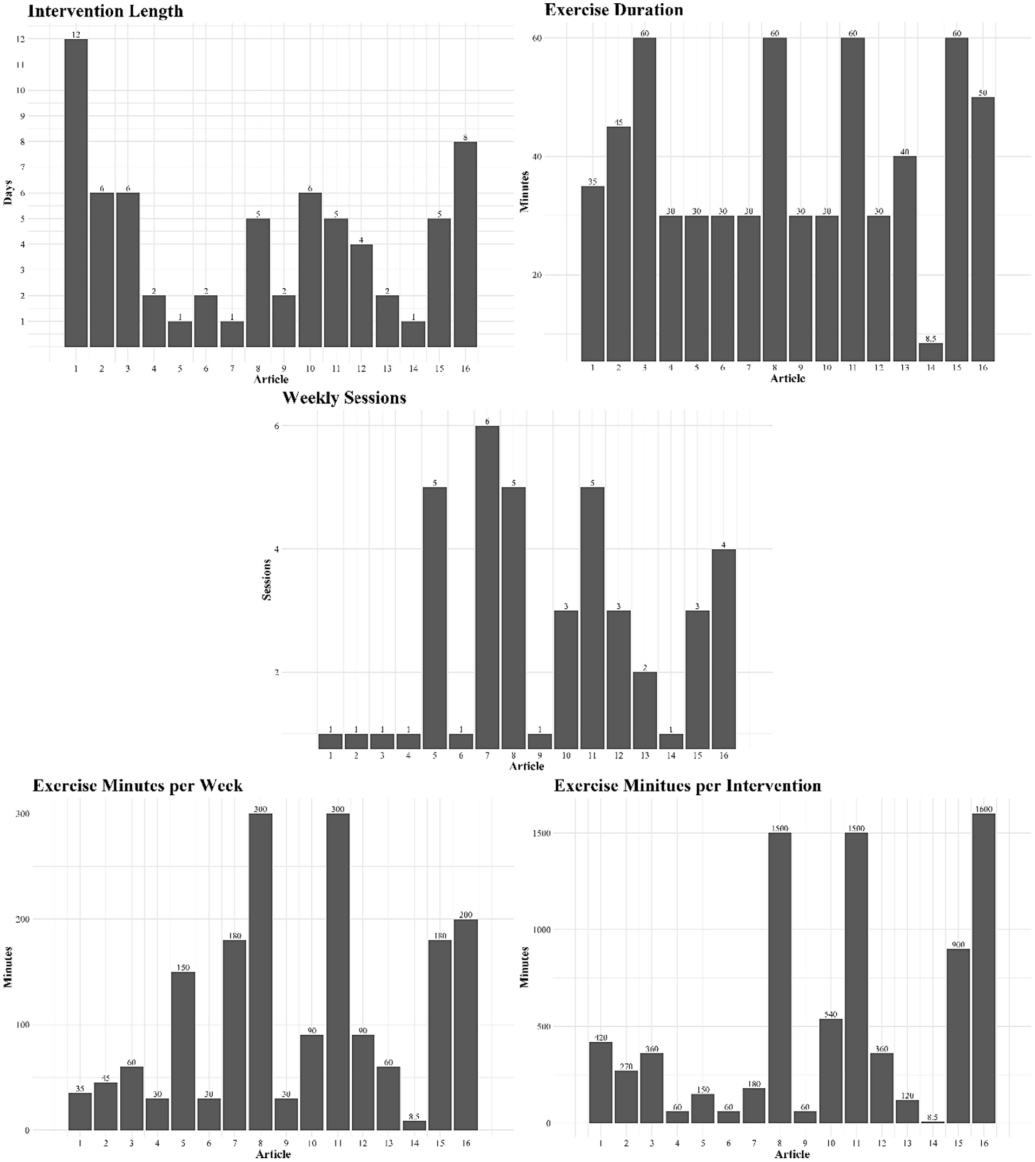
Intervention programs for each of the 16 articles, considering intervention length, exercise duration, weekly sessions, exercise minutes per week, and exercise minutes per intervention.

Based on the description of the games presented in the previous paragraph, it becomes evident that some are centered around sports, others focus on dance, and still others involve movement activities. In this context, to facilitate a more standardized analysis of interventions concerning the intervention program, three relevant pieces of information were extracted: the number of weeks of the intervention, the duration of each session, and the number of weekly sessions (Table 1, Figure 3) First, regarding the number of weeks of intervention, the studies had an average duration of 4.25 weeks (SD ± 3.02) with a minimum of 1 week and a maximum of 12 weeks. Regarding the duration of the session, an average duration of 44.67 min (SD ± 24.06 min) was observed, with a minimum of eight and a maximum of 1 h long. Finally, regarding the weekly frequency, we observed an average frequency of 2.5 times a week (SD ± 1.9) with a minimum of one single session and a maximum of six weekly sessions. Figure 3 also illustrates the exercise minutes per week and exercise minutes per intervention, values that were calculated based on the three pieces of information extracted from each article.

Lastly, considering the emotional measure the following aspects were evaluated: Anxiety - three articles; Attitude - one article; Depression - seven articles; Enjoyment - one article; Happiness - three articles; Health-Related QoL - three articles; Mood - one article; Motivation - one article; Optimism - one article; Patient Health - one article; Perceived Behavioral Control - one article; Perceived energy - one article; Relaxation - one article; Self-efficacy - one article; Self-esteem - one article; Stress - one article; Subjective Wellbeing - one article; Vigor - one article.

### Quality assessment results

As described in the method, each article was evaluated by two authors, considering 10 different aspects, each with a score from zero to 10 points. Then, an average score was evaluated from the evaluations of both authors and an average total score for each article. Figure 1 represents the distribution of the scores of the 10 evaluated aspects.

From the results, it was observed that the articles had an average total score of 84.34 points (SD ± 3.86) with a minimum score of 76.50 and a maximum of 89.50. Regarding the 10 criteria, the results indicate: (i) Relevance of the topic – 9.22 (SD ± 0.88; min 7; max 10); (ii) Methodological quality – 8.91 (SD ± 1.10; min 5.5; max 10); (iii) Sample size – 9.13 (SD ± 0.92; min 7.5; max 10); (iv) Statistical analysis – 8.53 (SD ± 1.58; min 4; max 9.50); (v) Reliability and validity of measurements – 8.59 (SD ± 1.19; min 5; max 10); (vi) Control of confounding variables – 6.47 (SD ± 2.11; min 4; max 10); (vii) Reproducibility and consistency – 8.97 (SD ± 0.64; min 8; max 10); (viii) Journal quality – 8.66 (SD ± 0.79; min 6.5; max 10); (ix) Conflict of interests - 6.75 (SD ± 3.85; min 0; max 10); (x) Practical and theoretical implications – 9.13 (SD ± 0.92; min 7; max 10).

As can be observed in the graph above, significant variances are noticeable between the works, albeit not being part of the initial method.

### Impact of Exergames training on emotional experience

As can be seen in Table 1, it was possible to notice that all works reported positive effects of the practice of Exergame, demonstrating that this modality of physical activity significantly and positively impacts aspects such as: Anxiety; Depression; Enjoyment; Happiness; Health-Related QoL; Mood; Optimism; Patient Health; Perceived Behavioral Control; Perceived energy; relaxation; Self-efficacy; Self-esteem; Stress; Subjective Wellbeing; and Vigor. Also, it was observed that the participant’s familiarity with the Exergame improved aspects such as Motivation and Satisfaction.

#### Anxiety, depression and stress

The studies measuring depression, anxiety, and stress utilized reputable quality questionnaires, including: Quick Inventory of Depressive Symptoms (QIDS); Patient Health Questionnaire-9 (PHQ-9); Hospital Anxiety and Depression Scale (HADS); Depression, Anxiety, and Stress Scale (DASS-21); Beck’s Depression Inventory (BDI); Center for Epidemiologic Studies Depression Scale (CES-D); Beck Anxiety Inventory (BAI); Health-Related Quality of Life (SF36); and The Parkinson’s Disease Questionnaire (PDQ-39). These questionnaires are recognized as quality measurements, demonstrating high reliability in the context of the studies and their findings.

The findings related to depression measurements in the studies are consistent, asserting that the practice of Exergames leads to a decrease in depression. These results were anticipated by most authors, as illustrated by the case of Rosenberg et al. (36). Out of nine studies that measured depression, only one did not demonstrate significant improvement in depression measures (44). Two of these studies indicate that Exergames achieved results comparable to traditional treatments for Parkinson’s disease and Multiple Sclerosis in terms of physical health measures, thereby reinforcing the validity of Exergames’ use in treatments for mobility-impaired patients (40, 42). In addition to this, Exergames exhibited superior depression and anxiety-related measurements compared to their traditional treatment counterparts, thus supporting the importance of Exergames in promoting mental health in treatments for physical health. This fact helps to strengthen the discussion about the importance of increasing evidence-based Parkinson’s disease treatment options for clinicians (42). Therefore, the application of Exergames can be more thoroughly examined in the clinical context, particularly within existing therapies for patients with motor dysfunction such as cycling for Multiple Sclerosis patients or physiotherapy for Hematopoietic Stem Cell Transplantation patients. This is due to its more pronounced antidepressant component compared to traditional exercises and warrants further investigation with mental disorder patients, as reflected in one study involving subthreshold depressive patients. Concerning measurements of anxiety and stress, two studies revealed a decrease in anxiety following approximately 30-min sessions of playing Exergames, observed in Hematopoietic Stem Cell Transplantation patients (40) and depressive older adults (36). Additionally, two other studies detected reductions in stress as well as anxiety within the same 30-min timeframe of Exergames engagement, specifically among healthy adults (43, 44). It is vital to underscore that these brief 30-min Exergames sessions appear sufficient to enhance mental health, as none of the studies reviewed herein indicated otherwise.

#### Positive affect

Beyond the mood variations previously detailed, certain studies have assessed positive affect attributes, encompassing enjoyment, happiness, and optimism. Concerning the modulation of enjoyment, two studies detected a notable increase following the engagement with Exergames, even within a brief one-week experiment (41, 48). This finding was also evident in post-stroke patients (41), likely contributing to their rehabilitation. In terms of enhancing happiness, three studies reported favorable outcomes. Two of these, conducted with adult participants and featuring analogous experimental conditions (30 min, one weekly session over 2 weeks) using the game “Your Shape: Fitness Evolved,” registered a substantial elevation in happiness levels (39, 43). In a study with children, Nguyen et al. (51) found that Exergames notably raised levels of happiness, displaying particularly pronounced results in children under weight management. Intriguingly, the same study did not detect a moderating effect on optimism levels (51), possibly hinting at a specific correlation between the enhancement in happiness and the weight control aspect of exergaming, without necessarily extending to future outlook and optimism regarding the benefits of practice.

#### Self-perception

Perceived behavioral control, self-efficacy, and self-esteem are facets of personal evaluation concerning an individual’s perceived capabilities. The findings related to perceived behavioral control reveal that engaging in Exergames, coupled with health messages about physical activity, can notably boost perceived behavioral control among children (37). This study also discovered that, in addition to enhancing perceived behavioral control, the game was efficacious in raising self-efficacy levels, even influencing weight management. Two other studies noted a positive effect of exergame participation on self-efficacy. Specifically, Li et al. (38) found that playing golf and bowling Exergames for 6 weeks significantly elevated self-efficacy among patients with subthreshold depression. Samendinger et al. (41) observed in healthy individuals that pre-intervention levels of self-efficacy were significant predictors of participant enjoyment and effort, thereby increasing adherence to exergame practice. Finally, a sole study reported a marked improvement in self-esteem due to playing the “Just Dance” game in both boys and girls (47). Interestingly, this study did not discern substantial differences in self-esteem enhancement between exergame practice and traditional physical education classes, thereby reinforcing the validity of exergame engagement.

#### Wellbeing and energy

Other vital components of the emotional experience include wellbeing and the awareness of bodily readiness. Several studies have explored these facets by examining various dimensions. For example, Huang et al. (43) found that perceptions of energy and relaxation significantly increased after playing Exergames, but this effect was only seen in participants who were already enthusiastic about exercise. Another related observation was the self-perception of vigor, which was noted both in adults (39) and children (47). Beyond these elements, the domains of wellbeing, chiefly associated with the subjective feeling of emotional wellness, and quality of life, primarily linked to the subjective awareness of physical wellness, were shown to be enhanced through the practice of Exergames involving dance (49) and sports (36), respectively. Together, these findings endorse the use of Exergames as a significant tool for enhancing emotional experiences, whether addressing short-term emotional states like stress, relaxation, and perceived energy, or long-term emotional traits such as anxiety, depression, and happiness.

## Discussion

The present work sought to systematically review the existing literature to investigate the impact of engaging in physical activity through Exergames on the emotional experience. The findings consolidated here collectively demonstrate that engaging in Exergames is associated with improvements in a wide range of aspects, including happiness, anxiety, depressive symptoms, mental health, health-related quality of life, self-worth, self-esteem, self-efficacy, perceived behavioral control, vigor, vitality, intrinsic motivation, perceived energy, and relaxation. Furthermore, although this study did not specifically aim to gauge the necessary intensity of Exergame practice or the optimal physical activity required to induce the desired emotional experience, it was evident that the effective practice of Exergames does not have to be highly intense to confer emotional benefits.

It is vital to underscore that the selection of articles involved a rigorous quality assessment, which revealed a high global average, indicating strong experimental control across the studies. Although some articles received lower scores in areas such as confounders and conflict of interest, it was decided to continue including them in the review. This decision was based on the understanding that these lower scores were often due to a lack of detailed description of these criteria, rather than evidence that these aspects were not appropriately controlled within the studies. Thus, the low scores do not necessarily imply that the articles failed to manage these criteria properly, but rather that they did not explicitly report how they were addressed.

In the topics below, we explore in more detail aspects such as the relationship between the findings and the emotional benefits of traditional physical activity, as well as the impact of the use of Exergames in everyday life and in clinical practice for the promotion of general health, well-being, and mental health.

### Impact of Exergames training on emotional experience

The selected articles that demonstrate the impact of Exergames on emotional experiences emphasize that Exergames can be beneficial for patients with depression (36, 46, 50). These findings are both reasonable and expected, given that physical activity has been proven to have highly beneficial effects on the symptoms of depression (52). It’s worth noting that using Exergames may be especially advantageous for patients who have limitations in physical activity due to mood disorders or avolition. In this context, the playful nature of Exergames can provide the necessary motivation to engage in activities, potentially leading to a positive impact on the emotional experience.

In addition to treating major depression, Exergames have shown promise in alleviating subthreshold depression, promoting positive emotions, enhancing self-efficacy (38), and improving not only feelings of depression but also anxiety and health-related Quality of Life (QoL) measures (40, 42, 43, 45). These findings highlight the potential for incorporating Exergames interventions into the multifaceted treatment approaches common in the psychiatric field. The benefits of Exergames are further supported by Randomized Controlled Trials (RCTs), which also indicate the possibility of reducing depression levels through exercise (53). This evidence positions Exergames as a valuable part of the overall treatment strategy for depression and related mental health conditions.

### Gaming and leisure activity

Although a recent article published in Nature Human Behavior argues that physical exercise does not significantly impact cognition (54), a substantial body of scientific research, including studies such as Warburton (2006), emphasizes the benefits of physical activities. This research supports the understanding that engaging in physical activity generally improves both the physical and emotional aspects of health in the majority of individuals. In light of this, the utilization of Exergames, a form of physical activity, is presented as an essential tool for enhancing and promoting public health, encompassing mental well-being. Exergames, in essence, are a specific type of physical activity and share many similarities with traditional forms of exercise. What distinguishes Exergames is their ludic characteristic, which refers to the gamification aspects incorporated into the activities (9, 10). As a result, most benefits derived from conventional physical exercise can also be attained through Exergames. According to the scientific findings from two studies reviewed here (42, 45), there is minimal difference in the physical impact symptoms between Exergames and traditional physical activities, especially in the reduction of symptoms in conditions like Parkinson’s disease or Multiple Sclerosis. Consequently, the gamification present in Exergames may lead to positive effects in areas such as adherence to exercise routines, satisfaction with the activities, and motivation to continue exercising.

These findings imply that Exergames can be leveraged as a tool to encourage and facilitate physical activities in daily life. This is particularly beneficial for individuals who struggle to maintain a routine of traditional physical exercises. Since Exergames can be pursued as a form of leisure, the enjoyment and entertainment aspects of gamification make them an appealing alternative (43). Thus, Exergames may offer a unique and engaging way to promote physical health, especially for those who find conventional exercise methods less motivating.

### Health promotion, public health, and clinical practice

As highlighted in the previous topics, the emotional benefits of engaging in physical activity through Exergames are strongly characterized by the ludic nature of the activity. This playful element typically enhances the willingness and interest of individuals to participate in physical activity (55, 56). Consequently, a form of exercise that not only promotes a marked improvement in the emotional experience but also fosters short- and long-term positive emotions becomes a valuable tool. Exergames are thus presented as a vital means to promote overall health and public well-being, demonstrating potential value for clinical application. In the context of public health, the collective findings presented here illustrate that the practice of Exergames is associated with emotional benefits across a diverse range of clinical groups. This includes Hematopoietic Stem Cell Transplantation patients (40), Hemodialysis patients (46), Parkinson’s patients (45), Depression patients (36), Overweight adults (50), and Post-stroke patients (48), among others. These results demonstrate that the external validity of Exergames can be generalized across multiple clinical demographics, underscoring their wide-reaching applicability. Moreover, the benefits extend to healthy participants of various age groups, encompassing children and adolescents (37, 47, 51), adults (39, 41–44), and older adults (38, 49). Concerning the safety of Exergames, it is worth noting that the studies reviewed here did not specifically aim to address this question, and thus, they are not characterized as phase I studies. However, among the research analyzed, no obvious or reported risks were observed. This indicates that, although there may be inherent risks of injury, as with any other physical activity, the practice of Exergames appears to be generally safe. Some studies even affirm the safety of Exergames (57–62). Nevertheless, it is essential to recognize that further studies are needed to assess the level of safety for different age groups and clinical conditions, to ensure a comprehensive understanding of the potential risks and benefits. An additional intriguing aspect of Exergames, particularly when considered as a therapeutic intervention, is their potential for remote administration. Unlike traditional therapies that may require patients to travel, Exergames allow for intervention to be performed from a distance. This flexibility can be particularly valuable for various patient groups. For example, a recent study demonstrated that Exergames were effectively utilized as a remote intervention for older adults with mild cognitive impairment or dementia (63). Exergames are emerging as an essential intervention tool in mental health treatment. Research has revealed their effectiveness in various domains, such as supporting the aging population (64), improving cognition and alleviating depression in older adults (63, 65), and serving as coping strategies for anxiety disorders during the COVID-19 quarantine period (66), as well as for generalized anxiety (67). Moreover, Exergames have been recognized as a broader tool for mental health treatments (68). When combined with the results reviewed in the present work, these findings reinforce the view that Exergames represent significant and meaningful interventions in mental health, relating both to cognitive processes and to clinical and subclinical emotional states. In essence, the practice of Exergames emerges as a suitable approach for public health interventions and strategies, serving both preventive mental health measures and therapeutic management.

### Limitations

In addition to the positive results, it is essential to acknowledge some limitations of the present review. Firstly, we recognize that the articles included in this study exhibit similarities regarding the practice of Exergames. However, it is worth noting that the list of 16 articles encompasses 12 different games, each with its unique characteristics in terms of intensity, type of movement, reinforcement systems (e.g., musical, image-based), and duration. The primary objective of this work was to present, for the first time, a comprehensive compilation of results from various studies investigating the impact of Exergames on the emotional experience. Despite this limitation, the results presented here are valid and support the need for future studies that delve deeper into these specific aspects of each game, potentially through a meta-analysis.

Secondly, based on the presented results and the general discussion above, it becomes apparent that the reported studies overwhelmingly demonstrate positive outcomes and an absence of negative results for the investigated measures. This finding raises the possibility of publication biases in this field of study, necessitating caution when interpreting the results. Moreover, this observation underscores the importance of replication studies to ascertain whether these results solely represent positive outcomes and the absence of negative ones.

Lastly, we conducted an assessment of the quality of the articles to ensure that the included works met the minimum criteria to support the review’s objectives. However, as evident from the results, there is significant variance among the studies in terms of their quality. As discussed earlier, these differences may be attributed to various factors, such as intervention characteristics, journal levels, sample sizes, and other publication-related aspects. Additionally, our review encompasses a relatively small sample of only 16 articles, which likely impacted the variance in our quality assessment results. To maintain the review’s focus and due to the limited number of included articles, we opted not to perform statistical analysis and inference on the extracted data. Although this decision may have limited our understanding of critical differences between the studies, it highlights the need for future reviews with a more substantial number of papers to explore these variations and their potential impact on result interpretation.

In conclusion, while this review offers valuable insights into the positive impact of Exergames on emotional experiences, it is essential to acknowledge the outlined limitations. By recognizing these constraints, researchers can better plan and execute future studies to build upon the existing knowledge in this field effectively.

## Conclusion

Our review supports the evidence that the practice of physical activity through Exergames generally generates an increase in positive emotions in the emotional experience. In this sense, the results found support both the use of Exergames as a leisure activity that promotes well-being and emotional regulation, as well as for health promotion, public health, and clinical practice purposes.

## Author contributions

LM conceived the initial idea. LM and PU designed the study. LM and SB selected and reviewed the articles and extracted the data. LM, PU, and SB drafted the manuscript article. All authors contributed to the article and approved the submitted version.

## Funding

Specifically, LM is supported by a postdoctoral research grant #21/05897-5, São Paulo Research Foundation (FAPESP). SB is supported by a postdoctoral research grant #20/08512-4, São Paulo Research Foundation (FAPESP).

## Conflict of interest

The authors declare that they have no known competing financial interests or personal relationships that could have appeared to influence the work reported in this paper.

The handling editor VO declared a part collaboration with the author PU.

## Publisher’s note

All claims expressed in this article are solely those of the authors and do not necessarily represent those of their affiliated organizations, or those of the publisher, the editors and the reviewers. Any product that may be evaluated in this article, or claim that may be made by its manufacturer, is not guaranteed or endorsed by the publisher.
